# TGFβ-activation by dendritic cells drives Th17 induction and intestinal contractility and augments the expulsion of the parasite *Trichinella spiralis* in mice

**DOI:** 10.1371/journal.ppat.1007657

**Published:** 2019-04-18

**Authors:** Nicola Steel, Aduragbemi A. Faniyi, Sayema Rahman, Stefanie Swietlik, Beata I. Czajkowska, Bethany T. Chan, Alexander Hardgrave, Anthony Steel, Tim D. Sparwasser, Mushref B. Assas, Richard K. Grencis, Mark A. Travis, John J. Worthington

**Affiliations:** 1 Faculty of Biology, Medicine and Health, University of Manchester, Manchester, United Kingdom; 2 Biomedical and Life Sciences, Faculty of Health and Medicine, University of Lancaster, Lancaster, United Kingdom; 3 The Lydia Becker Institute of Immunology and Inflammation, Faculty of Biology, Medicine and Health, University of Manchester, Manchester, United Kingdom; 4 Institute of Infection Immunology, TWINCORE, Center for Experimental and Clinical Infection Research, Hannover, Germany; 5 Faculty of Applied Medical Sciences, King AbdulAziz University, Jeddah, Saudi Arabia; 6 Wellcome Trust Centre for Cell-Matrix Research, University of Manchester, Manchester, United Kingdom; 7 Manchester Collaborative Centre for Inflammation Research, University of Manchester, Manchester, United Kingdom; University of California Riverside, UNITED STATES

## Abstract

Helminths are highly prevalent metazoan parasites that infect over a billion of the world’s population. Hosts have evolved numerous mechanisms to drive the expulsion of these parasites via Th2-driven immunity, but these responses must be tightly controlled to prevent equally devastating immunopathology. However, mechanisms that regulate this balance are still unclear. Here we show that the vigorous Th2 immune response driven by the small intestinal helminth *Trichinella spiralis*, is associated with increased TGFβ signalling responses in CD4+ T-cells. Mechanistically, enhanced TGFβ signalling in CD4+ T-cells is dependent on dendritic cell-mediated TGFβ activation which requires expression of the integrin αvβ8. Importantly, mice lacking integrin αvβ8 on DCs had a delayed ability to expel a *T*. *spiralis* infection, indicating an important functional role for integrin αvβ8-mediated TGFβ activation in promoting parasite expulsion. In addition to maintaining regulatory T-cell responses, the CD4+ T-cell signalling of this pleiotropic cytokine induces a Th17 response which is crucial in promoting the intestinal muscle hypercontractility that drives worm expulsion. Collectively, these results provide novel insights into intestinal helminth expulsion beyond that of classical Th2 driven immunity, and highlight the importance of IL-17 in intestinal contraction which may aid therapeutics to numerous diseases of the intestine.

## Author summary

Infection with intestinal parasitic worms is a major global health problem. We have therefore evolved means to drive the expulsion of these worms (known as helminths), based on protective (type 2) immune responses. However, if these immune responses are not regulated they can result in more harm than good. One protein that can be key in controlling immune responses is transforming growth factor beta (TGFβ). Using a model helminth which infects mice, we found that TGFβ was indeed signalling to the immune cells which can initiate the type 2 response, but rather than increasing the regulation of these T-cells it was driving a different inflammatory immune response (termed Th17). Interestingly, this Th17 response was important in expelling the parasite, as mice lacking the ability to activate the TGFβ protein, lacked Th17 responses and the ability to contract intestinal muscles and flush out the parasite. Our findings therefore provide new insights into how helminths are expelled and identify potential molecular targets for the prevention of helminth infection which affects billions of the world’s population in deprived communities.

## Introduction

Human intestinal helminths infect more than 1 billion of the world’s population, often affecting the most deprived communities [1]. These parasites are one of the most prevalent Neglected Tropical Diseases worldwide bringing huge morbidities to the host population; sub-Saharan Africa alone is estimated to lose 2.3 million disability-adjusted life-years annually [2]. Notwithstanding this hugely successful colonisation, we have evolved numerous Th2-driven mechanisms of parasite expulsion [3–8], which must be tightly regulated to avoid potential immunopathology, such as uncontrolled fibrosis and barrier dysfunction, as seen in ulcerative colitis [9].

The small intestinal helminth *Trichinella spiralis* is the leading causative agent of trichinosis, which globally exhibits burdens of around 12 million [10], equivalent to kinetoplastid-caused infections such as *Leishmania sp*. and *Trypanosoma cruzi* [11]. The life cycle consists of the release of larvae from nurse cells following pepsin digestion of contaminated meat in the stomach, prior to migration and swift development into adults in the small intestine. Male and female adults mate to produce new born larvae which migrate via the blood and lymph to the striated muscle where they form new nurse cells. Mouse models have demonstrated that infection produces a strong CD4+ T-cell [12,13] and type 2 cytokine [14–16] driven transient inflammation culminating in worm expulsion around day 15 post-infection (p.i.) in C57BL/6 mice. IL-9 driven mastocytosis [17] is key in *T*. *spiralis* expulsion [18–20], driving the degradation of epithelial tight junctions via the release of mast cell proteases during degranulation [21,22]. The resulting increase in luminal fluid, works in combination with Th2 driven alterations of enhanced intestinal propulsive activity. IL-13 and IL-4, signalling via signal transducer and activator of transcription factor 6 (STAT6) [23] on smooth muscle cells [24], allow jejunal muscle hypercontractility [23–25]. Despite the potential for immunopathology in terms of intestinal barrier weakening and exposure to luminal commensals, in combination these pathways produce the “weep and sweep’ mechanism [26], to drive out the enteric stage of infection with only short-lived pathology.

In comparison to other helminths, *T*. *spiralis* infection produces a robust Th2 response with evident pathology in terms of weight loss prior to intestinal worm expulsion [27], while the following encapsulation of new born larvae within the striated muscle is associated with a general malaise. Previous work has demonstrated the importance of the pluripotent cytokine TGFβ in the chronic muscular phase of the parasite life cycle [28], but the role of this complex cytokine during the intestinal phase remains unclear. Given the fundamental importance of TGFβ in regulating many aspects of T-cell biology [29] we chose to investigate the mechanistic function of TGFβ signalling in regulating the potential pathological immune response during *T*. *spiralis* enteric infection.

Here, we demonstrate that mice infected with *T*. *spiralis*, display enhanced TGFβ signalling in intestinal CD4+ T-cells which drives Th17 induction, as opposed to an increased regulatory T-cell (Treg) response. We find that the expression of integrin αvβ8 on dendritic cells (DCs), previously shown to be key in activating TGFβ and maintaining Tregs during intestinal homeostasis [30,31], is essential for the induction of TGFβ signalling in CD4+ T-cells and the generation of Th17 cells during infection. Importantly, mice lacking integrin αvβ8 on DCs (Itgb8 (Cd11c-cre)) have a delayed ability to expel the intestinal stage of the infection, despite an equivalent Th2 response to wild-type controls. Utilising the DEREG system for Treg ablation [32] demonstrates an essential requirement of Tregs for parasite expulsion, yet the adoptive transfer of Tregs into Itgb8 (Cd11c-cre) mice suggests that the reduced Treg level seen is not responsible for the delayed parasite expulsion in this model. Instead, we show that the Th17 response promotes intestinal contractility and the “sweep” mechanism of parasite expulsion. Our results therefore provide novel insights into the role of TGFβ during intestinal helminth infection, contributing greater understanding to mechanisms of helminth expulsion and potentially enteric diseases encompassing muscle hypercontractility.

## Results

### Small intestinal helminth infection with *Trichinella spiralis* results in increased TGFβ signalling in CD4+ T-cells, inducing Th17 rather than Foxp3+ regulatory T-cells

Expulsion of the small intestinal helminth *T*. *spiralis* is associated with a strong and acute T-helper 2 (Th2) CD4+ T-cell response, around one week p.i. in mice ([12–16] and S1A Fig). Mice develop a biphasic morbidity in parallel to the enteritis and myositis of infection [27], indicating a need to regulate this strong inflammatory response. We investigated the mechanistic role of the pluripotent cytokine TGFβ, which regulates many aspects of innate and adaptive immunity including T-cells [29], during the potential pathological immune response during *T*. *spiralis* enteric infection.

**Fig 1:**
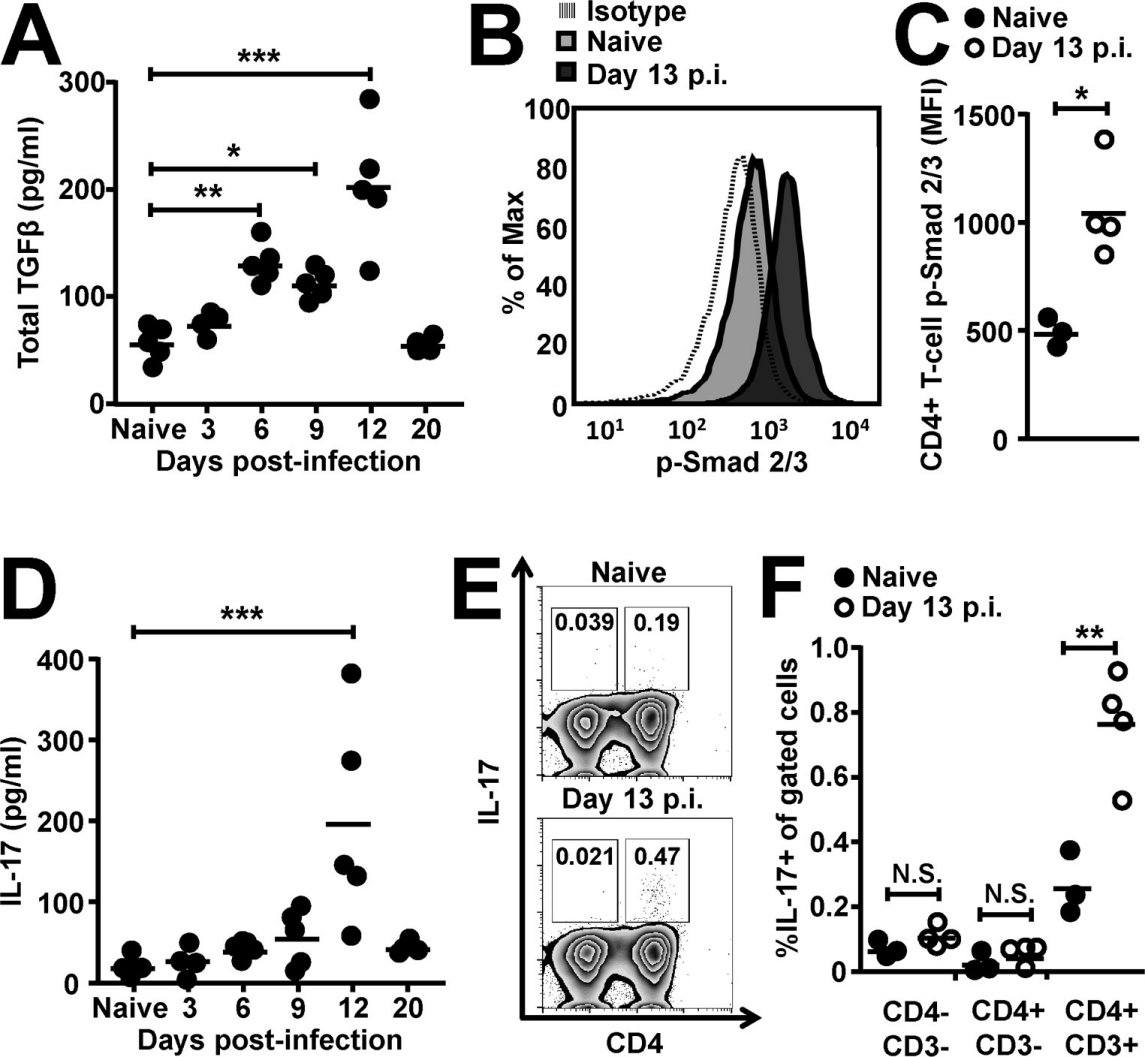
Infection with the small intestinal helminth *T*. *spiralis* increases TGFβ signalling in CD4+ T-cells producing late Th17 cell induction. Wild-type C57BL/6 mice were infected with 300 *T*. *spiralis* larvae and examined at the indicated time points. *(A)* Total TGFβ cytokine levels from *T*. *spiralis* antigen-stimulated mLN cells across the time-course of intestinal infection, determined via ELISA. *(B)* Representative flow cytometry plots and *(C)* mean fluorescence intensities for p-Smad 2/3 staining in small intestinal lamina propria CD4+ T-cells from uninfected and day 13 post-infected mice. *(D)* IL-17 cytokine levels from *T*. *spiralis* antigen-stimulated mLN cells across the time-course of intestinal infection, determined via cytometric bead array. *(E)* Representative flow cytometry plots of total CD45+ small intestinal lamina propria cells and *(F)* percentage IL-17 expression in small intestinal lamina propria CD4-CD3-, CD4+CD3- and CD4+CD3+ gated cells from uninfected and day 13 post-infected mice. Data (n = 3–5 mice per group) are from two independent experiments performed.*, P<0.05; **, P<0.01; ***, P<0.005; N.S., not significant via Dunnett’s multiple comparison following ANOVA *(A)* and *(D)* or student’s t-test *(C)* and *(F)* for the indicated comparisons between groups.

Wild-type C57BL/6 mice were infected with 300 *T*. *spiralis* larvae and followed throughout the time course of infection. We analysed parasite-specific cytokine production from mesenteric lymph node (mLN) cell preparations and saw a significant increase in TGFβ secretion in parallel to enhanced Th2 responses (IL-13, IL-9 and IL-4 production) at day 6 p.i. (Fig 1A and S1A Fig). Interestingly, in contrast to the reduction in IL-4, IL-9 and IL-13 cytokine release later in infection (S1A Fig), we saw a stronger, secondary peak of TGFβ at day 12 p.i. (Fig 1A). As TGFβ is produced as a latent cytokine requiring activation, we examined phosphorylation of Smad 2/3 (p-Smad2/3), which is the initial signalling event triggered by engagement of active TGFβ with its receptor. We saw significantly increased p-Smad2/3 levels in CD4+ T cells at day 13 p.i. in the small intestinal lamina propria (SILP) intestinal niche of the parasite (Fig 1B and 1C), indicating enhanced activation of TGFβ.

TGFβ signalling in CD4+ T-cells can result in the induction of Th17 [33–35], Th9 [36,37] or peripheral Treg subsets [38], depending on co-stimulatory signals and the surrounding cytokine milieu. Although we did not see any significant increase in IL-9 secretion at day 12 p.i. (S1A Fig), nor increase in the percentage of IL-9 expressing mLN CD4+ T-cells (S1B Fig) or Foxp3 expression in small intestinal CD4+ T-cells around this time-point (S1C and S1D Fig), we did see a significant increase in IL-17 secretion at day 12 p.i.in parallel to the secondary peak of TGFβ production (Fig 1D). This increase in IL-17 production was also concomitant with a significant increase in IL-6 (S1A Fig), which can synergise with TGFβ to drive Th17 cell induction [39]. Indeed, on performing intracellular flow cytometry we identified CD4+ cells as the source of the IL-17 produced during this infection (Fig 1E), with additional gating showing significant increases in IL-17 seen within the CD4+CD3+ T-cell gated population during infection (Fig 1F).

These data indicate that TGFβ signalling in CD4+ T-cells is induced during the enteric stage of *T*. *spiralis* infection and is associated with Th17 cell induction subsequent to the classical Th2 response.

### Expression of the TGFβ-activating integrin αvβ8 by DCs propagates TGFβ signalling in CD4+ T-cells and expulsion of enteric *T*. *spiralis* infection

**Fig 2:**
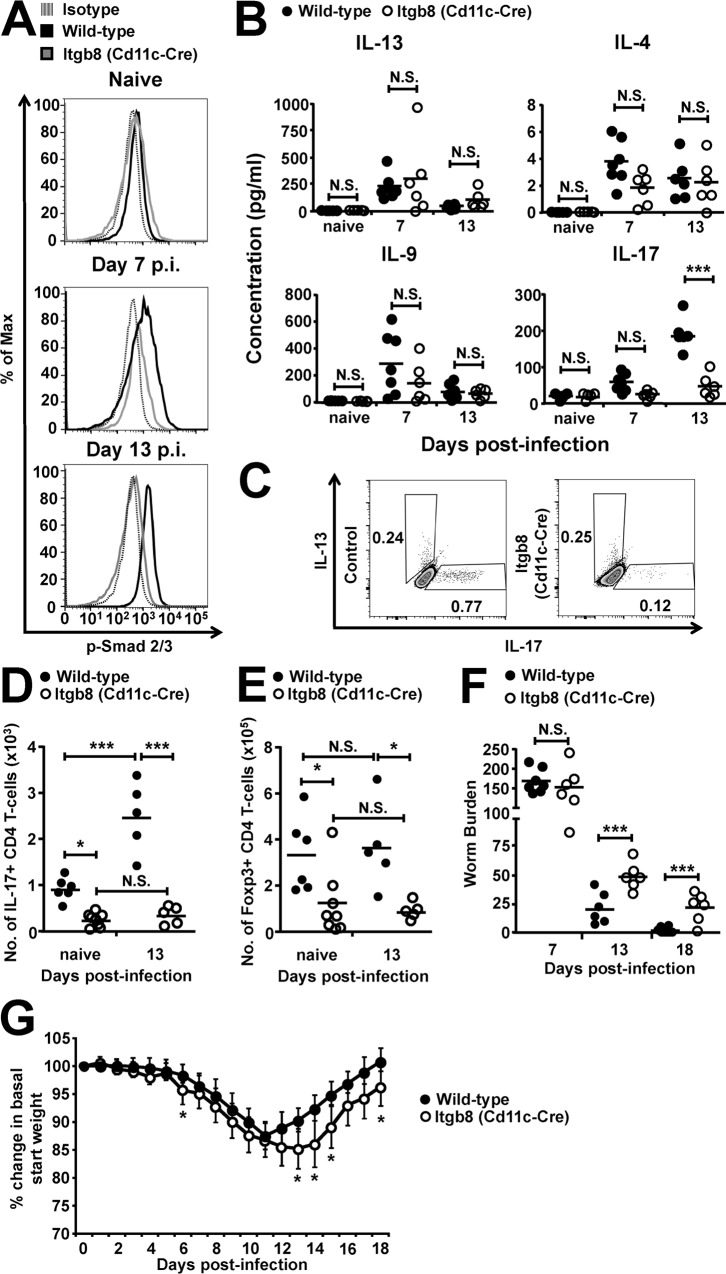
Mice lacking the TGFβ-activating integrin αvβ8 on DCs have delayed expulsion of the small intestinal helminth *T*. *spiralis*. Wild-type and *Itgb8 (CD11c-cre)* mice were infected with 300 *T*. *spiralis* larvae and examined at the indicated time-points post-infection. *(A)* Representative flow cytometry plots for p-Smad 2/3 staining in small intestinal lamina propria CD4+ T-cells. *(B)* IL-13, IL-4, IL-9, and IL-17 cytokine levels from *T*. *spiralis* antigen-stimulated mLN cells from wild-type and *Itgb8 (CD11c-cre)* mice, determined via ELISA. *(C)* Representative flow cytometry plots for intracellular IL-17 and IL-13 expression in small intestinal lamina propria CD4+ T-cells isolated from wild-type and *Itgb8 (CD11c-cre)* mice at day 13 post-infection. Number of *(D)* IL-17+ and *(E)* Foxp3+ CD4 T-cells in the small intestinal lamina propria of wild-type and *Itgb8 (CD11c-cre)* mice, assessed via flow cytometry. *(F)* Worm burdens from wild-type and *Itgb8 (CD11c-cre)* mice at days 7, 13 and 18 p.i. *(G)* Percentage change in basal start weight in wild-type and *Itgb8 (CD11c-cre)* mice over the course of infection. Data (n = 6–10 mice per group) are from two independent experiments performed. *, P<0.05; ***, P<0.005; N.S., not significant via Bonferonni’s multiple comparison following ANOVA *(B)*, *(D)*, and *(E)* or student’s t-test *(F)*and *(G)* for the indicated comparisons between groups.

**Fig 3:**
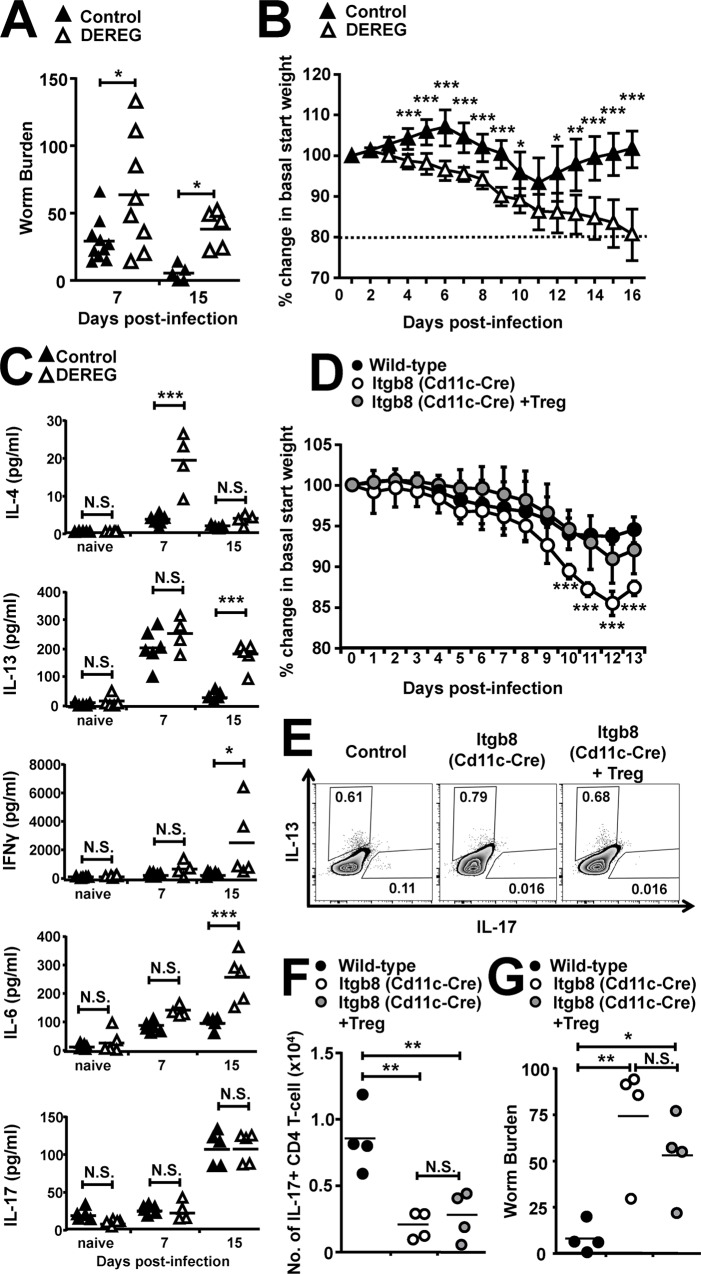
Depletion of Foxp3+ Tregs during *T*. *spiralis* infection results in extreme morbidity and delayed helminth expulsion, but the immune kinetics and delayed expulsion seen in mice lacking the TGFβ-activating integrin αvβ8 on DCs are independent of Tregs. DEREG mice were treated every 2 days with 200 ng diphtheria toxin or PBS (Control) 2 days prior to infection with 300 *T*. *spiralis* larvae and examined at the indicated time-points post-infection. *(A)* Worm burdens from control and DEREG mice at days 7 and 15 following infection. *(B)* Percentage change in basal start weight in control and DEREG mice during time course of infection, dashed line indicates point of morbidity sacrifice threshold. *(C)* IL-4, IL-13, IFNγ, IL-6 and IL-17 cytokine levels from *T*. *spiralis* antigen-stimulated mLN cells from control and DEREG mice at different time-points post-infection, determined via CBA. Data (n = 4–11 mice per group) are from two independent experiments performed. Wild-type, *Itgb8 (CD11c-cre)* and *Itgb8 (CD11c-cre)* mice adoptively transferred with 1x10^6^ Tregs were infected with 300 *T*. *spiralis* larvae 2 days following cell transfer and examined at the indicated time-points post-infection. *(D)* Percentage change in basal start weight in wild-type, *Itgb8 (CD11c-cre)* and *Itgb8 (CD11c-cre)* mice adoptively transferred with Tregs during time course of infection. *(E)* Representative flow cytometry plots for intracellular IL-17 and IL-13 expression in mLN CD4+ T-cells isolated from wild-type, *Itgb8 (CD11c-cre)* and *Itgb8 (CD11c-cre)* mice adoptively transferred with Tregs, at day 13 post-infection. *(F)* Number of IL-17+ CD4 T-cells in the mLN of wild-type, *Itgb8 (CD11c-cre)* and *Itgb8 (CD11c-cre)* mice adoptively transferred with Tregs, at day 13 post-infection, assessed via flow cytometry. (*G)* Worm burdens from wild-type, *Itgb8 (CD11c-cre)* and *Itgb8 (CD11c-cre)* mice adoptively transferred with Tregs, at day 13 following infection. Data (n = 4 mice per group) are from two independent experiments performed.*, P<0.05; **, P<0.01; ***, P<0.005; N.S., not significant via Bonferonni’s multiple comparison following ANOVA *(C)*, *(F) and (G)*, and student’s t-test *(A)*, *(B)* and *(D)* for indicated comparisons between groups.

We next sought to determine the mechanisms responsible for enhanced TGFβ signalling during *T*. *spiralis* infection. The requirement for the activation of latent TGFβ prior to function [40] led us to investigate the potential for integrin αvβ8, a key activator of latent TGFβ in the intestine expressed by dendritic cells (DCs) [30,31,41], to be responsible for the enhanced signalling seen in CD4+ T-cells. To this end, we analysed T-cell responses following infection with 300 *T*. *spiralis* larvae in mice lacking integrin αvβ8 on DCs (Itgb8 (CD11c-Cre) mice [30]) and wild type littermate controls. We found that the increase in TGFβ signalling observed in CD4+ T-cells during *T*.*spiralis* infection was significantly reduced in Itgb8 (CD11c-Cre) mice, with pSmad2/3 levels remaining similar to those observed in uninfected mice (Fig 2A). Interestingly, this lack of TGFβ signalling in CD4+ T-cells did not affect the classical Th2, Th9 nor Th1 immune cytokine responses during the time-course of infection, with no significant difference observed in parasite specific IL-13, 4, 9 (Fig 2B) and IFNγ (S2A Fig) production from mLN antigen restimulation. This was also reflected in the similar IgG response seen at day 18 post-infection (S2B Fig), which is a key indicator of Th1/2 balance, and IL-9 expression in mLN CD4+ T-cells at day 13 post-infection (S2C Fig). However, IL-17 production was significantly reduced at day 13 p.i., in both mLN restimulations (Fig 2B) as well as from small intestinal lamina propria CD4+ T-cells (Fig 2C), which were also observed to produce similar IL-13 levels (Fig 2C). Indeed, beyond the previously reported initial baseline differences in intestinal Th17 cells in Itgb8 (CD11c-Cre) mice ([30] and (Fig 3D)), total small intestinal lamina propria IL-17+ CD4+ T-cell numbers failed to significantly increase following infection at day 13 p.i. in Itgb8 (CD11c-Cre) mice as compared to wild-types (Fig 2D). Interestingly, we also observed a significant reduction in small intestinal lamina propria Foxp3+ regulatory T-cells at rest in the Itgb8 (CD11c-Cre) mice, with neither wild-type or Itgb8 (CD11c-Cre) mice Treg numbers altering during enteric *T*. *spiralis* infection (Fig 2E). Thus, during enteric *T*. *spiralis* infection, enhanced TGFβ activation by integrin αvβ8 on DCs is important in triggering infection-induced TGFβ signalling pathways in CD4+ T-cells, driving Th17 cells, and maintaining Treg numbers during homeostasis.

Strikingly, and despite the maintained Th2 and Th9 response in Itgb8 (CD11c-Cre) mice, we observed a significant delay in worm expulsion and exacerbated weight loss (Fig 2F and 2G) following infection, as compared to wild-type mice. This delay was not associated with differences in other proposed mechanisms involved in helminth expulsion, with no significant difference in crypt/villus architecture (S2D Fig), goblet cell hyperplasia [42] (S2E Fig), mastocytosis [18–20] and associated MMCP-1 production [21,22] (S2F and S2G Fig) or RELMβ expression [43] (S2H Fig) between wild-type and Itgb8 (CD11c-Cre) mice. Collectively these data indicate that despite the maintenance of a Th2 response in Itgb8 (CD11c-Cre) mice, TGFβ activation by integrin αvβ8 on DCs is essential for triggering TGFβ signalling pathways in CD4+ T-cells and promoting parasite expulsion.

### Foxp3+ Tregs are required for efficient *T*. *spiralis* worm expulsion, but their adoptive transfer does not rescue Th17 cell numbers or helminth expulsion in mice lacking the TGFβ-activating integrin αvβ8 on DCs

We next focussed on uncovering the mechanisms responsible for the delayed expulsion of the small intestinal helminth *T*. *spiralis* from mice lacking the TGFβ activating integrin αvβ8 on DCs. Given the stark baseline reduction in small intestinal Foxp3+ Tregs in Itgb8 (CD11c-Cre) mice (Fig 2E), we utilised the DEREG mouse model, which allows specific ablation of Foxp3+ Tregs by injection of diphtheria toxin [32], to directly test the functional role of Foxp3+ Tregs during infection. DEREG mice treated with diphtheria toxin had successful complete depletion of Foxp3-GFP+ cells during the time course of the experiment, although we did see grow back of non-GFP Foxp3+ cells (S3A Fig), which have previously been demonstrated to possess no inhibitory function [44]. We found that worm burdens in DEREG mice recapitulated the delayed expulsion seen in Itgb8 (CD11c-Cre) mice, with significantly increased worm burdens observed at day 7 and 15 p.i. (Fig 3A). Furthermore, as in Itgb8 (CD11c-Cre) mice, a heightened weight loss was apparent, but this took on differing kinetics, with mice presenting with sustained significant weight loss from day 4 p.i. in DEREG mice versus day 13 p.i. in Itgb8 (CD11c-Cre) mice (Fig 3B versus Fig 2G). Moreover, this weight loss in infected DEREG mice did not recede, despite attempts to rehydrate the animals with saline, resulting in mice reaching the threshold for humane end-point and the cessation of the experiments at day 15 p.i.(Fig 3B).

To try and decipher reasons behind this extreme morbidity, we examined parasite-specific cytokine responses following mLN antigen restimulation. In stark contrast to Itgb8 (CD11c-Cre) mice, we observed significant increases in IL-4 production at day 7 p.i.; while IL-13 and IFNγ increased at day 15 p.i. (Fig 3C). Interestingly no differences were seen in parasite–specific IgG antibody nor MMCP-1 production, as compared to untreated control mice, indicating no overall imbalance in the Th1/Th2 paradigm (S3B and S3C Fig). Recent publications have discovered an essential role for Foxp3+ Tregs in eliminating the small intestinal helminth *Heligmosoides polygyrus*, with Treg depletion associated with delayed worm expulsion following an uncontrolled “cytokine storm” [45]. We therefore looked at other pro-inflammatory cytokines and we did indeed see a significant increase in IL-6 at day 15 p.i. (Fig 3C). Importantly, we did not see the reduction in IL-17 later in infection in DEREG mice, as seen in Itgb8 (CD11c-Cre) mice (Fig 2B vs. Fig 3C).

Despite the clear evidence demonstrating a complete lack of Tregs could mediate worm expulsion and weight loss during *T*. *spiralis* infection, we next asked if the adoptive transfer of Tregs to Itgb8 (CD11c-Cre) mice was sufficient to rescue worm expulsion kinetics. Despite the successful restoration of small intestinal lamina propria Foxp3+ cells (S3D and S3E Fig) resulting in augmented percentage weight (Fig 3D), we saw no alteration in IL-13 or IL-17 production in Treg treated Itgb8 (CD11c-Cre) mice (Fig 3E and 3F), culminating in similar delayed expulsion as in untreated Itgb8 (CD11c-Cre) mice (Fig 3G).

Collectively, these data suggest Foxp3+ Tregs are an important cell type in the context of *T*. *spiralis* infection and are required for efficient expulsion of small intestinal helminths via inhibiting runaway inflammation, as well as modulating weight loss pathology. However, given the increased Th1 and Th2 cytokines but maintenance of IL-17 production in the DEREG system and the failure of Treg adoptive transfer to rescue Itgb8 (CD11c-Cre) delayed worm expulsion, this mechanism seems not to be solely responsible for the phenotype displayed in *T*. *spiralis* infected Itgb8 (CD11c-Cre) mice.

### IL-17 drives intestinal muscle hypercontractility during *T*. *spiralis* infection

Given that the adoptive transfer of Tregs into Itgb8 (CD11c-Cre) mice restored weight loss kinetics but not worm expulsion, coupled with the strong Th2 response and accompanying effector mechanisms seen in infected Itgb8 (CD11c-Cre) mice, we next examined a role for the altered Th17 cell population during this infection. We hypothesised that IL-17 may influence muscle hypercontractility rather than mastocytosis induced luminal fluid increases, hence the “sweep” but not the “weep” aspect during expulsion of the enteric phase of *T*. *spiralis*.

**Fig 4:**
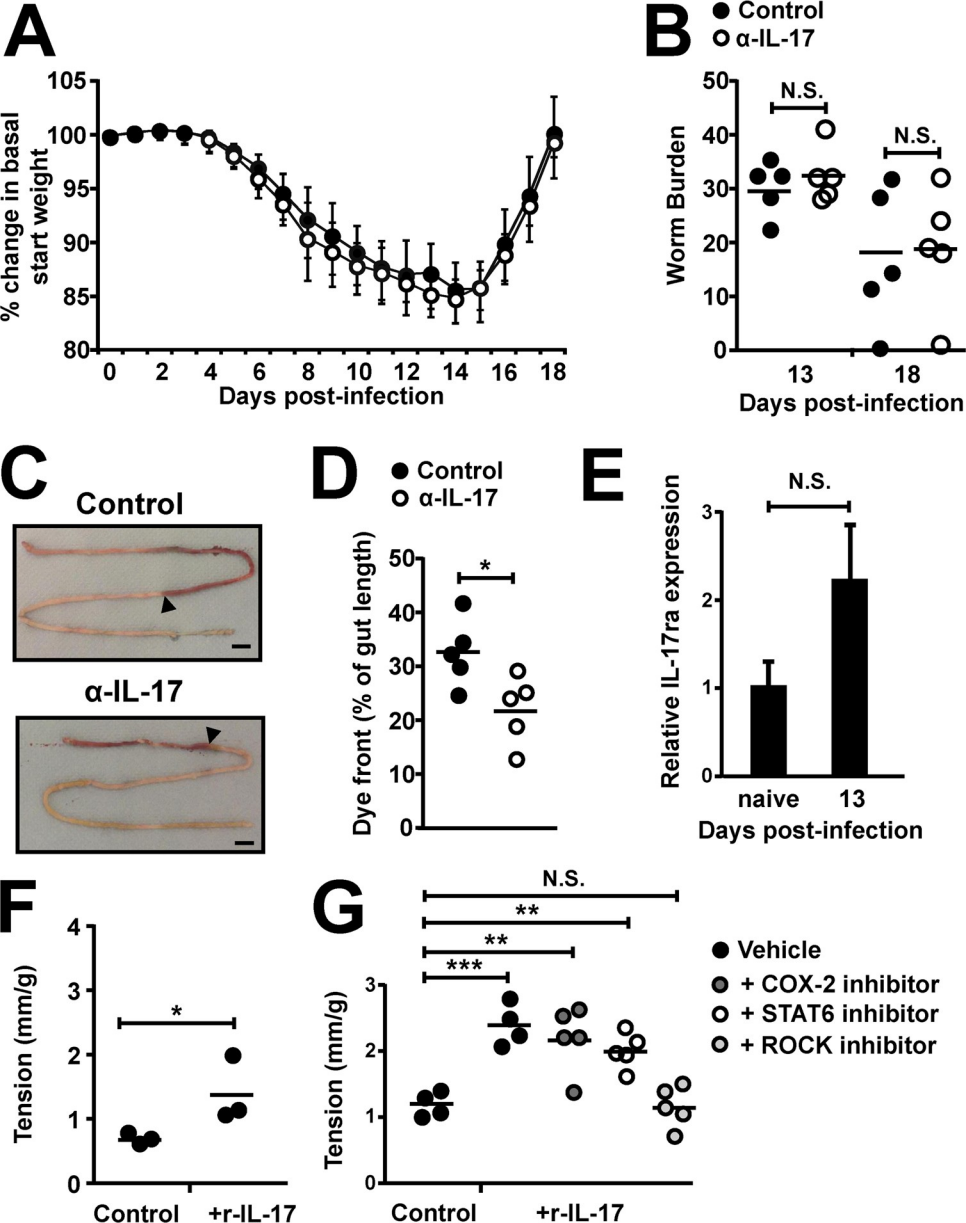
IL-17 drives intestinal muscle hypercontractility during *T*. *spiralis* infection and *ex vivo* via the ROCK signalling pathway. C57BL/6 mice were infected with 300 *T*. *spiralis* larvae and treated with 100μg of anti-IL-17 or control antibody (Bio-X-Cell) every 3 days from day 7 post-infection. *(A)* Percentage change in basal start weight in control and α-IL-17 treated mice over the course of infection. *(B)* Worm burdens from control and α-IL-17 treated mice at days 13 and 18 p.i. Chow was removed 12 hrs prior to sacrifice at day 13 and mice received 200μls carmine red in methylcellulose 20 minutes before sacrifice. *(C)* Representative macroscopic images, arrow indicates front of dye and scale bar = 1 cm, and combined data *(D)*. Data (n = 5 mice per group) are from two independent experiments performed. *(E)* Expression of IL-17ra in isolated jejunal muscle layer at rest and day 13 p.i, via qPCR relative to HPRT housekeeping gene. *(F)* Isolated jejunal strips from C57BL/6 wild-type mice were incubated in media with/without the addition of 10ng/ml rIL-17 for 6 hours prior to measuring longitudinal muscle tension generated in response to carbachol (10^-6^M) in an isolated tissue bath and *(G)* with/without the prior addition of the COX-2 and ROCK inhibitors celecoxib (10μM) and Y-27632 (10μM) and STAT6 inhibitor AS1517499 (100nm). Data (n = 3–5 mice per group) are from two independent experiments performed. *, P<0.05; **, P<0.01; ***, P<0.005; N.S., not significant via Bonferonni’s multiple comparison following ANOVA *(G)* and student’s t-test *(A)*, *(B)*, *(D)*, *(E)* and *(F)* for indicated comparisons between groups.

To investigate the individual importance of IL-17 in *T*. *spiralis* infection, we blocked IL-17 from day 7 p.i. in C57BL/6 mice via antibody depletion. Although we saw no significant difference in weight or worm burdens when IL-17 was depleted from day 7 p.i. (Fig 4A and 4B), we did see a significant reduction in *in vivo* transit time in the small intestine, as measured by the transit of orally gavaged carmine dye (Fig 4C and 4D). Importantly, the depletion of IL-17 did not impinge on the CD4+ mLN T-cell production of IL-13 (or IFNγ) (S4A and S4B Fig), suggesting that alterations in transit time were possibly due to the absence of IL-17, rather than a follow-on effect of reduced Th2 cytokines known to induce small intestinal hypercontractility [23,24]. We next isolated jejunal smooth muscle and confirmed the expression of the IL-17ra via qPCR both at rest and following infection with *T*. *spiralis* (Fig 4E). This suggested the potential for IL-17 to directly influence intestinal smooth muscle contraction.

To investigate this hypothesis, we first incubated isolated jejunal strips of intestine from wild-type mice with or without rIL-17 prior to assessing longitudinal muscular tension *ex vivo* generated in response to stimulation with carbachol. Treatment with rIL-17 produced a significant increase in tension (Fig 4F), indicating IL-17 could promote tension and therefore potentially drive parasite expulsion. We next asked what downstream pathways could be responsible for transposing the IL-17 signal, with COX-2 and STAT6 pathways previously being shown to drive TGFβ and IL-4/13 intestinal contraction respectively, following *T*.*spiralis* infection [24,46]. To this end, we repeated *ex vivo* contraction experiments with prior exposure to inhibitors for both pathways, but detected no alteration in the hypercontraction response to carbachol following rIL-17 incubation (Fig 4G). Previous studies have demonstrated that Rho kinase signalling is emerging as an important mediator of intestinal smooth muscle contraction [47], with IL-13 and TNFα driving smooth muscle contraction via the small GTPase, RhoA via STAT6 and NF-κβ signalling respectively [48]. We therefore targeted the RhoA downstream effector kinases via prior exposure to a ROCK pathway inhibitor, and observed an inhibition of the ability of IL-17 to produce significant hypercontraction in response to carbachol (Fig 4G).

Collectively, these data show that, although not solely sufficient for worm expulsion or altered weight loss, IL-17 has direct effects on small intestinal hypercontractility, acting via the ROCK signalling pathway, and could potentially be responsible for the delayed expulsion seen in *T*. *spiralis* infected Itgb8 (CD11c-Cre) mice.

### rIL-17 treatment following *T*. *spiralis* infection rescues intestinal muscle hypercontractility and worm expulsion in mice lacking the TGFβ-activating integrin αvβ8 on DCs

**Fig 5:**
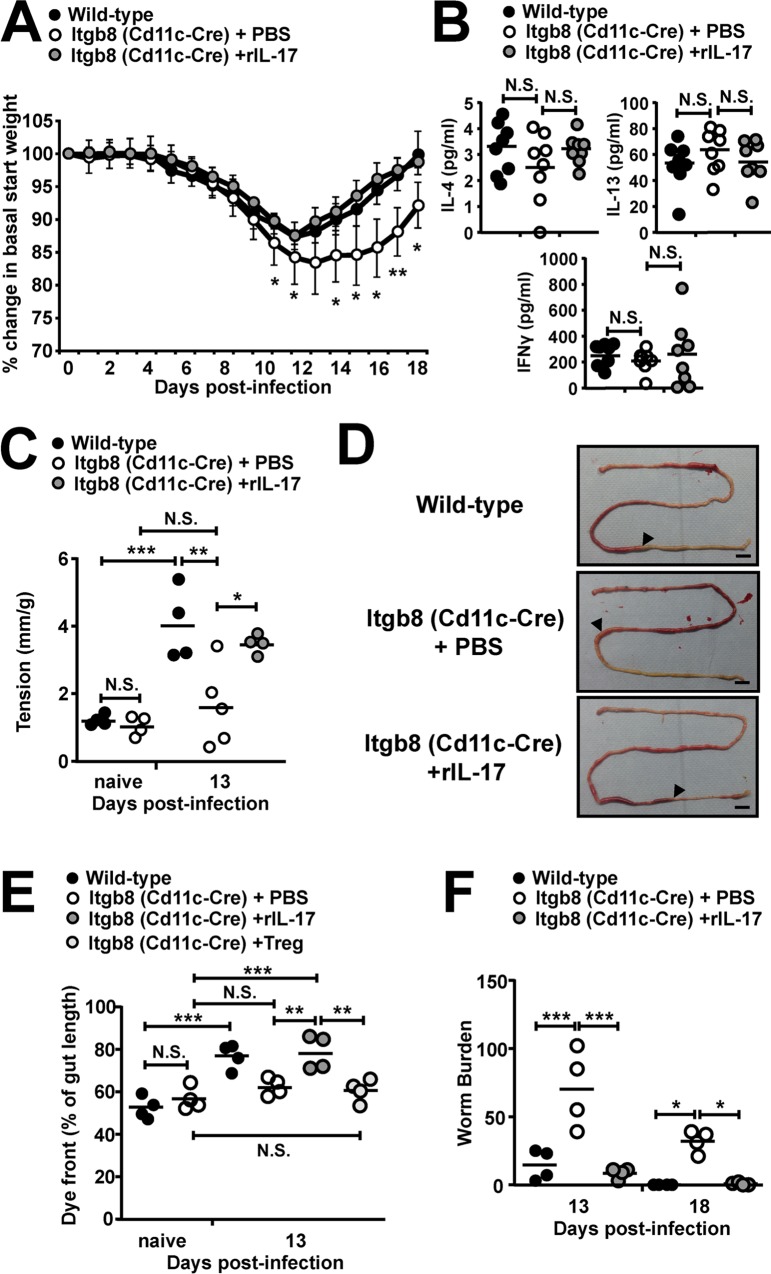
rIL-17 treatment following *T*. *spiralis* infection restores worm expulsion in mice lacking the TGFβ-activating integrin αvβ8 on DCs via rescuing intestinal muscle hypercontractility. Wild-type and *Itgb8 (CD11c-cre)* mice were infected with 300 *T*. *spiralis* larvae and treated with PBS or 2ug of recombinant IL-17 every 3 days from day 9 post-infection and examined at the indicated time-points post-infection. *(A)* Percentage change in basal start weight in wild-type and *Itgb8 (CD11c-cre)* PBS or rIL-17 treated mice over the course of infection. *(B)* IL-4, 13 and IFNγ cytokine levels from *T*. *spiralis* antigen-stimulated mLN cells at day 13 post-infection, determined via ELISA. *(C)* Jejunal longitudinal muscle tension generated in response to carbachol (10^-6^M) from wild-type and *Itgb8 (CD11c-cre)* mice PBS or rIL-17 treated, intestinal contraction was examined in an isolated tissue bath at time points indicated. Wild-type and *Itgb8 (CD11c-cre)* mice were infected with 300 *T*. *spiralis* larvae and treated with PBS, 2ug of recombinant IL-17 every 3 days from day 9 post-infection or adoptively transferred with 1x10^6^ Tregs 2 days prior to infection and examined at the indicated time-points post-infection. Chow was removed 12 hrs prior to sacrifice at day 13 and mice received 200μls carmine red in methylcellulose 20 minutes before sacrifice. *(D)* Representative macroscopic images, arrow indicates front of dye and scale bar = 1 cm, and combined mean data of dye front *(E)*. *(F)* Worm burdens from wild-type and *Itgb8 (CD11c-cre)* PBS or rIL-17 treated mice at days 13 and 18 following infection. Data (n = 4–8 mice per group) are from two-three independent experiments performed. *, P<0.05; **, P<0.01; ***, P<0.005; N.S., not significant via Bonferonni’s multiple comparison following ANOVA *(B)*, *(C)*, *(E)* and *(F)* and student’s t-test *(A)* for indicated comparisons between groups.

Given the role of IL-17 in driving small intestinal contraction, we tested whether the reduced levels of parasite specific IL-17 production seen in Itgb8 (CD11c-Cre) mice were responsible for delayed worm expulsion via a reduced small intestinal hypercontractility. To this end, we examined if we could rescue delayed expulsion in these mice via treatment with recombinant IL-17. Treatment with rIL-17 from day 9 p.i. completely restored the weight loss kinetics (Fig 5A) to levels seen in wild-type mice. This rescue of weight loss following rIL-17 treatment was not associated with any changes in parasite-specific IL-4, IL-13 or IFNγ cytokine production (Fig 5B), nor in parasite specific IgG responses (S5A Fig).

Next, we examined isolated longitudinal muscle tension between jejunal samples from wild-type and Itgb8 (CD11c-Cre) mice. Although there was no differences in tension either at baseline nor following carbachol treatment in naïve mice (S5B Fig and Fig 5C), following infection Itgb8 (CD11c-Cre) mice failed to significantly increase jejunal tension in response to stimulation with carbachol at day 13 p.i., as seen in in wild-type infected mice (Fig 5C and [23–25]). Moreover, the treatment of infected Itgb8 (CD11c-Cre) mice with rIL-17 rescued this muscular tension to wild-type levels *ex vivo* (Fig 5C). Next, we examined *in vivo* contraction in the small intestine and despite no alteration at base line (Fig 5E and S5C Fig), we saw significantly delayed transit time following infection in Itgb8 (CD11c-Cre) mice, which was again rescued via the addition of rIL-17, but could not be restored by the adoptive transfer of Tregs (Fig 5D and 5E). Strikingly, in parallel to this recued small intestinal contraction, treatment with rIL-17 from day 9 p.i. completely restored the worm burden kinetics in infected Itgb8 (CD11c-Cre) mice (Fig 5F) to levels seen in wild-type mice.

In sum, these data indicate that TGFβ activation by integrin αvβ8 on DCs is essential for triggering TGFβ signalling pathways in CD4+ T-cells allowing the maintenance of Tregs and induction of Th17 cells during *T*. *spiralis* infection. Tregs play a key role in mediating weight loss and aiding helminth expulsion via inhibiting runaway inflammation, while Th17 produced IL-17 contributes to enhanced muscular “sweep” tension promoting parasite expulsion.

## Discussion

We have evolved immune driven mechanisms to allow the expulsion of intestinal helminths, with the “weep and sweep” supplied by increased intestinal epithelial permeability and muscle contraction [21–25] essential during *T*. *spiralis* infection. In most cases these expulsion mechanisms rely on Th2 cytokines resulting in minimal host damage indicating an essential role for regulation to avoid immunopathology; however the pathways and mechanisms involved remain unclear. Our data now indicate an essential role for TGFβ, activated via DC expressed integrin αvβ8, in parasite expulsion via the maintenance of Tregs and induction of Th17 cells, as opposed to simply immuno-regulation. Using the small intestinal dwelling helminth *T*. *spiralis*, we observed increased TGFβ signalling in CD4+ T-cells and production of Th17 cells late in infection. Mechanistically, we find that enhanced TGFβ signalling in T-cells occurs via expression of the TGFβ-activating integrin αvβ8 on DCs and that DC-specific lack of this integrin results in increased weight loss and delayed worm expulsion, despite the occurrence of the “classical” Th2 response. The total ablation of Tregs, in the DEREG model, demonstrates a role for this cell in aiding helminth expulsion via inhibiting runaway inflammation, while their adoptive transfer into Itgb8 (CD11c-Cre) mice indicates a key role in mediating infection induced weight loss. Moreover, Itgb8 (CD11c-Cre) mice lack intestinal hypercontractility that can be rescued via treatment with recombinant IL-17, fully restoring both weight loss and worm expulsion kinetics. We have therefore identified a novel, non-Th2 based, mechanistic pathway that could potentially be targeted to treat helminth infection and contractile diseases of the intestine.

Previously, TGFβ signalling within T-cells has been shown to play an important role in downregulating Th2 responses via downregulation of the key transcription factor GATA-3 [49,50]. Indeed, we have previously shown that enhanced TGFβ signalling in T-cells during chronic Th1-induced *Trichuris muris* infection also occurs via expression of the TGFβ-activating integrin αvβ8 on DCs. Moreover the lack of this integrin on DCs completely protects mice from *T*. *muris* infection due to an enhanced protective Th2 response in this model of large intestinal infection [51]. However, here, we did not see any alteration in parasite-specific Th2 responses associated with delayed parasite expulsion, nor any increase in IFNγ production in *T*. *spiralis* infected Itgb8 (CD11c-Cre) mice. These data may represent tissue-specific effects of TGFβ activation in the small and large intestine, or more likely that it is mechanistically difficult to surpass the robust Th2 driven cytokine response seen during a normal *T*. *spiralis* infection.

Instead we saw a lack of IL-17 production at day 13p.i. in mice lacking the TGFβ-activating integrin αvβ8 on DCs, accompanying an unaltered Th1/Th2 balance. ILC3s are known as important producers of IL-17 at mucosal barriers [52]; however, it appeared that the IL-17+ population was found within the CD3/CD4+ T-cell pool, therefore likely bona-fide Th17 cells. Increased TGFβ release is seen in human DCs following treatment with *T*. *spiralis* antigen [53], although these DCs go on to favour a Th2 rather than a Th17 response, indicating that other cellular populations or subsets are producing cytokines which favour Th17 induction during *in vivo* infection.

Along with TGFβ, numerous cytokines are involved in Th17 induction, including IL-6, IL-21, IL-1β and IL-23 (reviewed in [39]). The production of IL-6 specifically at day 13p.i. is likely to be driving the Th17 induction [54] and possibly explains why we saw minimal IL-17 production corresponding with the initial peak of TGFβ at day 6 p.i. The source of IL-6 remains elusive, but Th17 induction via DC produced TGFβ relies on IL-6 production from a CD301b DC population during intranasal infection [55], indicating a possible DC source. Overall, it will be interesting to define what cytokines and from which cells are involved in inducing the Th17 seen during *T*. *spiralis* infection. Furthermore, it is interesting to postulate the antigen specificity in the system. The data displayed are based on parasite-specific cytokine responses as well as PMA/ionomycin re-stimulation and, given helminths directly influence the intestinal microbiome [56,57], it remains to be seen if Th17 responses to bacterial antigens would influence the outcome to *T*. *spiralis* infection.

Our initial hypothesis to explain the delayed parasite expulsion was based on the previous finding that TGFβ-activating integrin αvβ8 is key in Treg development, as mice lacking the integrin on DCs have reduced Foxp3+ Tregs within the colonic lamina propria [30]. We therefore predicted that a possible reduction in Tregs in the small intestine of Itgb8 (CD11c-Cre) mice could be playing a role in the delayed expulsion seen during *T*. *spiralis* infection. Indeed, recent publications have demonstrated a requirement for Tregs for efficient helminth expulsion in the small intestinal *H*. *polygyrus* model [45]. Of note previous findings have demonstrated that *H*. *polygyrus* produces a TGFβ mimic which acts as an immunomodulatory agent aiding chronicity [58], while our results suggest host TGFβ promotes expulsion of *T*. *spiralis*, as in our hands *T*. *spiralis* antigens have no TGFβ like properties [59]. This disparity could possibly be explained by the differing tissue localisation of the helminths during establishment, sub-mucosal versus epithelial niches or the local cytokine milieu, as *H*. *polygyrus* infection suppresses IL17 production [60]. However, the demonstration of reduced Tregs within the small intestinal lamina propria of Itgb8 (CD11c-Cre) mice, coupled with the delayed expulsion and increased weight loss in Treg depleted DEREG mice was initially indicative that reduced Treg numbers were solely responsible for the phenotype seen in Itgb8 (CD11c-Cre) mice. However, the extreme morbidity and mixed cytokine production observed, with no difference in IL-17 production, supported the previous hypothesise of “immunological chaos” in these mice. These results, coupled with the failure to rescue intestinal hypercontractility and worm expulsion kinetics when Itgb8 (CD11c-Cre) had been successfully adoptively transferred with Tregs, pointed towards additional mechanisms involved in *T*. *spiralis* delayed expulsion in Itgb8 (CD11c-Cre) mice. Adoptive transfer of Tregs was sufficient to return weight loss to wild-type levels, which has previously been shown to be mediated by the peptide hormone cholecystokinin [27]. It will therefore be of interest to examine any potential for Tregs to interact with production of cholecystokinin from enteroendocrine cells, given the recent interest in the immunoendocrine axis [61].

We have recently identified activated Tregs as expressing the TGFβ-activating integrin αvβ8 [62] which in the presence of IL-6 allows Tregs to induce Th17 cells in a GARP-dependent process [63]. It was therefore possible that the reduced small intestinal Treg numbers seen in Itgb8 (CD11c-Cre) mice were also responsible for the reduction in Th17 induction during *T*. *spiralis* infection. However, given that Treg depleted DEREG mice still mounted similar IL-17 responses as infected controls and the adoptive transfer of Tregs into Itgb8 (CD11c-Cre) mice failed to rescue Th17 numbers, the delayed parasite expulsion and reduced Th17 induction appears independent of Treg activation of TGFβ, and directly dependent on DCs.

We began to examine several other mechanisms of helminth expulsion, and saw no changes in goblet cell kinetics or mastocytosis. Mucosal mast cells are also under the control of TGFβ, with the cytokine controlling mast cell expression of the gut homing integrin alphaE and MMCP-1 [64], essential for the weep aspect of *T*. *spiralis* expulsion^20, 21^. It is therefore surprising that both mastocytosis and release of MMCP-1 appeared normal in Itgb8 (CD11c-Cre) mice. This may reflect alternative cell-specific mechanisms for the activation of TGFβ, with the active cytokine signalling within the local cellular environment, such as the T cell synapse via DC expressed αvβ8. This hypothesised high level of control is perhaps unsurprising given the multiple pathways that TGFβ drives. Indeed, previous studies have demonstrated that epithelial expression of the TGFβ –activating integrin αvβ6 is essential for mast cell hyperplasia and MMCP-1 release during small intestinal helminth infection [65]. Moreover, epithelial cell specific αvβ6 null mice demonstrated abnormal mastocytosis and MMCP-1 expression [66] linked with reduced expression of the intestinal homing integrin alphaE [67]. Collectively, this supports the context specific integrin activation of TGFβ, allowing distinct and tight control of this pleiotropic cytokine.

Finally, after we observed rIL-17 treatment was able to rescue weight loss and expulsion kinetics in *T*. *spiralis* infected Itgb8 (CD11c-Cre) mice, we investigated the possibility for IL-17 driving parasite expulsion. Indeed, late acting Th17 cells would prove beneficial in aspects of immunity and repair to helminth infection, with IL-17 driving Paneth cell antimicrobial peptide production [68] and IgA secretion [69]. This may be another important role of Th17 induction during *T*. *spiralis* infection, as microbial dysbiosis is a hallmark of intestinal helminth infection [57] and the microbiota also plays important roles in Th17 cell induction [39]. Although the data presented here was gained from co-housed littermate controls, it is interesting to speculate on how the microbiome may alter intestinal contraction via the induction of Th17 cells. Alternatively, IL-17 can have direct effects on nematode behaviour [70] and epithelial permeability; TGFβ activation by αvβ8 integrin has been shown to be important for increased alveolar permeability in acute respiratory distress syndrome [71]. Although we saw no changes at the microscopic level in infected Itgb8 (CD11c-Cre) mice, including goblet cells and RELMβ expression, Th17 production of IL-22 is related to goblet cell hyperplasia and enhanced worm expulsion [72]. Taking these potential mechanisms into account, and given the minimal effect of extra-intestinal larvae on muscle function at this timepoint [73], we examined the possibility of alterations in jejunal contractility as a possible role for the delayed expulsion, concentrating on a possible role for IL-17 as an expulsion mechanism.

Gut contraction during *T*. *spiralis* infection has previously been shown to be driven by Th2 cytokines and TGFβ, acting via STAT6 and COX-2 respectively [24,46]. Although we saw no changes in Th2 responses in our model, the reduced gut levels of active TGFβ seen in infected Itgb8 (CD11c-Cre) mice, could be involved directly in the reduced contraction seen. However, we observed a significant effect of rIL-17 on baseline gut contraction, reinforcing data from other investigators [74], that was independent of COX-2, as well as a complete rescue during infection by the addition of rIL-17, but not Tregs; making it unlikely that TGFβ was directly responsible for contractility differences. Previous studies have demonstrated that Rho kinase signalling is emerging as an important mediator of intestinal smooth muscle contraction [47], and may play a role during pathophysiology [75]. Moreover, there is precedent within the mucosal barrier of the lung, for αvβ8 dependent Th17 induction driving smooth muscle contraction via NF-κβ and the ROCK2 signalling cascade, with Itgb8 (CD11c-Cre) mice protected from airway hyper-responsiveness in response to house dust mite and ovalbumin sensitization and challenge [76]. Indeed, inhibiting the ROCK pathway, rather than STAT6, prevented hypercontractility of small intestinal muscle in response to IL-17 indicating a potential similar mechanism *ex vivo*. However, it remains likely that Th2 cytokines and IL-17 may interact during the intestinal hypercontractility response to *T*. *spiralis* infection *in vivo*, with IL-17 previously shown to enhance IL-13 driven STAT6 intracellular responses in mouse and human lung epithelial cells [77].

Collectively, these data support a novel role for IL-17 in driving the intestinal contraction and augmenting the expulsion of *T*. *spiralis*. The inhibition of IL-17 during *T*. *spiralis* infection in wild-type mice further supports a key role for this cytokine in infection induced hypercontractility, but it must be noted that worm expulsion was unaltered when compared to vehicle treated animals. These data, when coupled with the complete rescue of weight, contractility and worm expulsion seen in IL-17 treated Itgb8 (CD11c-Cre) mice, suggests an additional facet, possibly reduced intestinal Tregs, that further promotes the key role of IL-17 within the Itgb8 (CD11c-Cre) model. An important question remains as to what regulates the strong Th2 response seen during *T*. *spiralis* infection. Although we did see some increased morbidity in terms of weight loss during the infection of Itgb8 (CD11c-Cre) mice, our adoptive transfer experiments suggest this is most likely due to the decreased Treg population and possibly the increased worm burden phenotype seen. As discussed earlier, activation of TGFβ via other mechanisms in a cell specific context may be responsible, or it may be a combination of several factors; as seen by the dual roles of IL-10 and TGFβ seen in *T*. *spiralis* nurse cell immunopathology [28]. Indeed IL-10 has previously been shown to be essential in avoiding fatal immunopathology in response to the microbiota during another epithelial dwelling helminth, *Trichuris muris* [78]. Tregs are likely to play a role, and are often associated with helminth infection, but we are reliant on more subtle approaches to remove distinct Treg subsets, as our results confirm global depletion as being detrimental to mouse survival by failing to regulate the majority of inflammatory pathways [45].

In summary, we have highlighted an important cellular and molecular pathway by which the DC expressed TGFβ-activating integrin αvβ8, maintains intestinal Tregs and drives the induction of Th17 cells late during infection with the small intestinal helminth *T*. *spiralis*. Tregs are essential for mediating infection induced weight loss, while the resulting Th17 produced IL-17 mediates the contraction of jejunal muscle via ROCK signalling aiding the “weep and sweep” mechanism of helminth expulsion. Thus, we have identified the molecular mechanism maintaining Tregs and driving Th17 induction and helminth expulsion, beyond the classical Th2 responses. Additionally, whether the Th17 pathway can be harnessed therapeutically in other parasitic diseases or pathologies encompassing muscle hypercontractility should be a focus of further studies.

## Materials and methods

### Animals

C57BL/6 mice were purchased from Harlan Laboratories. Mice lacking integrin αvβ8 on DCs via expression of a conditional floxed allele of β8 integrin in combination with CD11c-Cre (Itgb8 (CD11c-Cre) mice) [30] and DEREG mice [32], all on a C57BL/6 background, have been previously described and were bred in house. For Itgb8 (CD11c-Cre) mice transgene negative littermate controls were used in all experiments. For DEREG mice transgene positive littermates were treated with PBS for controls. All experiments were on male, age-matched mice maintained in specific pathogen-free conditions at the University of Manchester and used at 6 to 12 weeks of age.

### Ethics statement

All animal experiments were performed under the regulations of the Home Office Scientific Procedures Act (1986), specifically under the project licence PPL 40/3633. The project licence was approved by both the Home Office and the local ethics committee of the University of Manchester. Animal euthanasia occurred using approved schedule 1 methods.

### *Trichinella spiralis* infection

The maintenance, infection and recovery of *T*. *spiralis* were carried out as previously described [79]. Mice were orally infected with 300 larvae and individually weighed on a daily basis. Worm burdens were assessed by counting the number of worms present in the small intestine as described previously [79].

### Treg and IL-17 depletion and treatment

Foxp3+ Tregs were depleted in DEREG mice as described [32], via i.p. injection of 200 ng diphtheria toxin (Merck) every 2 days from 2 days prior to infection. IL-17 was blocked via i.p. injection of 100μgs of anti-IL-17α (17F3) or IgG1 isotype control (MOPC-21) (BioXCell) from day 7 p.i. and every 3 days following. For Treg treatment, cells were isolated via Treg isolation kit (Miltenyi) according to manufacturer’s instructions. Cells were assessed as >95% Foxp3+ and mice were adoptively transferred with 1x10^6^ Tregs prior to infection. For IL-17 treatment, 2ug of recombinant IL-17 (Peprotech) was injected i.p. every 3 days from day 9 post-infection. In both gain of function treatments control animals received PBS vehicle injections at identical time points.

### Flow cytometry staining

Spleens and mesenteric lymph nodes (mLNs) were removed from mice and disaggregated through a 100 μm sieve. Small intestines were excised and lamina propria lymphocytes (SILP) were prepared essentially as described [80] with slight modification in the tissue digestion step (digestion medium used was RPMI with 10% Foetal calf serum, 0.1% w/v collagenase type I and Dispase II (both Invitrogen), and tissue was digested for 30 min at 37°C). Cell suspensions were blocked with anti-FcγR antibody (clone 24G2; eBioscience) before labelling with antibodies specific for CD3 (eBio500A2), CD4 (clone GK1.5; eBioscience), Foxp3 (clone FJK-16s; eBioscience), IL-13 (clone eBiol13A; eBioscience), IFNγ (clone XMG1.2; eBioscience), IL-17(eBio17B7; eBioscience), IL-9 (RM9A4e; Biolegend) or p-Smad 2/3 (Santa Cruz). For intracellular cytokine analysis cells were incubated for 12 hours with 1x Cell stimulation cocktail (plus protein inhibitors) (ebioscience). Cells were then stained with antibodies using the eBioscience Foxp3 permibilization kit according to the manufacturer's instructions. For pSmad2/3 staining, an Alexa Fluor 594-labelled donkey anti-goat secondary antibody was used (Invitrogen). All samples were analysed on a FACS LSRII.

### Cell re-stimulation

mLN and SILP cells were prepared as described above before incubating with 50μg/ml *T*. *spiralis* antigen for 24 hours in media (RPMI-1640, 10% FCS, 100U/ml Pen/strp, 5%NEAA, L-glutamine and HEPES, 0.05 mM β-mercaptoethanol (SIGMA)). Cell-free supernatants were analysed for cytokine production via cytometric bead array (BD) or paired ELISA antibodies (anti-IFNγ, clone XMG1.2 and R4-6A2; anti-IL-13, clone eBio13A and eBio1316H; anti-IL-4, clone 11B1and BVD6-2462, anti-IL-17 clone eBio17CK15A5 and eBio17B7; (eBioscience)). For TGFβ analysis samples were acid-activated prior to detection on a mouse TGF-beta 1 DuoSet ELISA (R and D Systems).

### Histology

Intestinal tissue was fixed in Carnoy’s solution and embedded in wax prior to mast or goblet cell staining via toludine blue or Schiff's reagent, respectively. Following antigen retrieval, RELMβ was labelled via primary antibody 1:400 (Abcam-ab11429) followed by detection with an Elite ABC HRP Kit (Vectastain) according to manufacturer’s instructions. After mounting, positive cells were enumerated in 20 randomly selected villus crypt units (VCU) and results presented as mean number of positive cells/20 VCU (± S.D.). Lengths of villus/crypts were enumerated via image J.

### Serum antibody and MMCP-1

Serum was obtained from blood at the time of sacrifice via centrifugation at 15000×g. Parasite specific IgG1 and IgG2a assessed via 5 μg/ml *T*. *spiralis* antigen coated ELISA plates in 0.05 M carbonate/bicarbonate buffer, pH 9.6. IgG1 and IgG2a were detected using biotinylated rat-anti mouse antibodies (Pharmingen, UK and Serotec, UK respectively) diluted in PBS-Tween and visulaised using streptavidin peroxidase and ABTS substrate prior to being read 405nm on a VersaMax microplate reader (Molecular devices, UK). Mouse mast cell protease-1 assessed via ELISA according to manufacturer’s instructions (Moredun).

### Intestinal contraction

*Ex vivo* intestinal contraction was measured as previously described [81]. Briefly, 3cm isolated jejunal strips were placed in oxygenated (95%O_2_-5%CO_2_) Krebs solution and surgical silk was used to hang the tissue longitudinally in an isolated tissue bath (Radnoti). Tissues were equilibrated for 30mins at 37°C under tension (1g), prior to baseline and carbachol (10^-6^M) response readouts being measured. The maximum force generated by the tissue was assessed (AD Instruments and Labchart Reader 8) and expressed in milligrams after normalising for cross sectional area [81]. In some cases, jejunal tissue was incubated in 10ng/ml rIL-17 for 6 hours in medium (Leibovitz’s L-15, 10% FCS, 100 U/ml Pen/strep, 50mg/ml gentamicin, 5% NEAA, L-glutamine and HEPES, 0.05 mM β-mercaptoethanol),following 2 hour treatment with 10μM celecoxib (COX-2 inhibitor), 100nM AS1517499 (STAT6 inhibitor) or 10uM Y-27632 (ROCK inhibitor) (Sigma) prior to measuring longitudinal muscle tension generated in response to carbachol (10^-6^M).

*In vivo* intestinal contraction was assessed via a 12 hour fast prior to gavage of 200μl of 6% carmine red dye (Sigma) in 0.5% methylcellulose 400c.p. (Sigma) before measuring distance of dye front, confirmed via tissue blotting, and gut length precisely 20mins later.

### Quantitative polymerase chain reaction

Total RNA was purified from small intestinal isolated jejunal muscle strips using Trizol reagent according to the manufacturer’s instructions (ThermoFischer). RNA was reverse transcribed using oligo(dT) primers and complementary DNA for specific genes detected using a SYBR Green qPCR Kit (Roche). Gene expression was normalized to HPRT levels. IL-17ra Forward-5’ CAAGTTTCACTGGTGCTGCC; IL-17ra Reverse-5’ TAGTCTGCAACTGGCTTGGG; HPRT Forward-5’ GCGTCGTGATTAGCGATGATGAAC; HRPT Reverse-5’ GAGCAAGTCTTTCAGTCCTGTCCA.

### Statistics

Results are expressed as mean ± S.D.. Where statistics are quoted, two experimental groups were compared via the Student’s t test for non-parametric data. Three or more groups were compared with ANOVA, with Dunnett’s or Bonferroni’s post-test as indicated. A p value of <0.05 was considered statistically significant. *, P<0.05; **, P<0.01; or ***, P<0.005 for indicated comparisons, error bars represent SD of means.

## Supporting information

S1 Fig Infection with the small intestinal helminth *T*. *spiralis* does not increase Th9 or Foxp3+ regulatory T-cells at day 13 post-infection. Wild-type C57BL/6 mice were infected with 300 *T*. *spiralis* larvae and examined at the indicated time points. *(A)* IL-4, 13, 6 and 9 cytokine levels from *T*. *spiralis* antigen-stimulated mLN cells across the time-course of intestinal infection, determined via cytometric bead array. *(B)* Representative flow cytometry plots of percentage IL-9 expression in mLN CD4+ T-cells from uninfected and day 13 post-infected mice. *(C)* Representative flow cytometry plots and *(D)* Percentage Foxp3 expression in small intestinal lamina propria CD4+ T-cells from uninfected and day 13 post-infected mice. Data (n = 3–5 mice per group) are from two independent experiments performed. *, P<0.05; **, P<0.01; ***, P<0.005; N.S., not significant via Dunnet’s multiple comparison following ANOVA *(A)* or student’s t-test *(D)* for the indicated comparisons between groups.
(TIF)

S2 Fig Mice lacking the TGFβ-activating integrin αvβ8 on DCs demonstrate no alterations in parasite specific antibody, small intestinal goblet or mast cell kinetics following infection with the helminth *T*. *spiralis*. Wild-type and *Itgb8 (CD11c-cre)* mice were infected with 300 *T*. *spiralis* larvae and examined at the indicated time-points post-infection. *(A)* IFNγ cytokine levels from *T*. *spiralis* antigen-stimulated mLN cells from wild-type and *Itgb8 (CD11c-cre)* mice, determined via ELISA. *(B)* Parasite-specific serum IgG1 and IgG2a levels in wild-type and *Itgb8 (CD11c-cre)* mice at day 18 post-infection. *(C)* Number of IL-9+ CD4 T-cells in the mLN of wild-type and *Itgb8 (CD11c-cre)* mice at day 13 p.i., assessed via flow cytometry. *(D)* Villus/crypt lengths assessed via examination of 20 randomly selected VCU in wild-type and *Itgb8 (CD11c-cre)* mice following infection, quantified via ImageJ software. Number of *(E)* goblet and *(F)* mast cells/20 VCU accessed via periodic acid-Schiff’s and toluidine blue histology staining respectively from wild-type and *Itgb8 (CD11c-cre)* mice. *(G)* Serum MMCP-1 levels from wild-type and *Itgb8 (CD11c-cre)* mice following infection, obtained via ELISA. *(H)* RELMβ+ cells/20VCU from wild-type and *Itgb8 (CD11c-cre)* mice assessed via immunohistochemistry. All data (n = 4–10 mice per group) are from two independent experiments performed.*, P<0.05; **, P<0.01; ***, P<0.005; N.S., not significant via Bonferonni’s multiple comparison following ANOVA *(A)*, *(D)*, *(E-H)* or student’s t-test *(B)*,and *(C)* for the indicated comparisons between groups.
(TIF)

S3 Fig Successful depletion of Foxp3+ Tregs during *T*. *spiralis* infection results in no parasite-specific antibody or mastocytosis differences, while adoptive transfer of Tregs restores the small intestinal lamina propria population in *Itgb8 (CD11c-cre)* mice. DEREG mice were treated every 2 days with 200 ng diphtheria toxin or PBS (Control) 2 days prior to infection with 300 *T*. *spiralis* larvae and examined at the indicated time-points post-infection. *(A)* The percentage of Foxp3+ CD4 T-cells in the mLN, as assessed via flow cytometry antibody staining and/or Foxp3-GFP reporter. *(B)* Parasite-specific serum IgG1 and IgG2a levels in Control and DEREG mice at day 15 post-infection, obtained via ELISA. *(C)* Serum MMCP-1 levels from Control and DEREG mice following infection, obtained via ELISA. Data (n = 4–9 mice per group) are from two independent experiments performed. Wild-type, *Itgb8 (CD11c-cre)* and *Itgb8 (CD11c-cre)* mice were adoptively transferred with 1x10^6^ Tregs were infected with 300 *T*. *spiralis* larvae 2 days following cell transfer. Representative flow cytometry plots *(D)* and *(E)* percentage Foxp3 expression in small intestinal lamina propria CD4+ T-cells from day 13 post-infection. Data (n = 4 mice per group) are from two independent experiments performed. **, P<0.01; ***, P<0.005; N.S., not significant via Dunnet’s multiple comparison following ANOVA *(A) and (E)*, Bonferonni’s multiple comparison following ANOVA *(C)* and student’s t-test *(B)* for indicated comparisons between groups.
(TIF)

S4 Fig Ablation of IL-17 during *T*. *spiralis* infection does not alter CD4+ T-cell IL-13 response. C57BL/6 mice were infected with 300 *T*. *spiralis* larvae and treated with 100μg of anti-IL-17 or control antibody (Bio-X-Cell) every 3 days from day 7 post-infection. *(A)* Number of mLN IFNγ and IL-13 positive CD4+ T-cells and *(B)* representative flow cytometry plots. Data (n = 5 mice per group) are from two independent experiments performed. N.S., not significant via student’s t-test for indicated comparisons between groups.
(TIF)

S5 Fig Mice lacking the TGFβ-activating integrin αvβ8 on DCs do not have baseline differences in intestinal muscle contraction and rIL-17 treatment following *T*. *spiralis* infection does not alter parasite specific antibody responses. Wild-type and *Itgb8 (CD11c-cre)* mice were infected with 300 *T*. *spiralis* larvae and treated with PBS or 2ug of recombinant IL-17 every 3 days from day 9 post-infection and examined at the indicated time-points post-infection. *(A)* Parasite-specific serum IgG1 and IgG2a levels in wild-type and *Itgb8 (CD11c-cre)* PBS or rIL-17 treated mice at day 18 following infection, obtained via ELISA. *(B)* Base line jejunal longitudinal muscle tension in naïve wild-type and *Itgb8 (CD11c-cre)* mice in an isolated tissue bath. Chow was removed 12 hrs prior to sacrifice at day 13 and mice received 200μls carmine red in methylcellulose 20 minutes before sacrifice. *(C)* Representative macroscopic images of wild-type and *Itgb8 (CD11c-cre)* naïve mice, arrow indicates front of dye and scale bar = 1 cm. Data (n = 4 mice per group) are from two independent experiments performed. N.S., not significant via Bonferonni’s *(A)* multiple comparison following ANOVA and student’s t-test *(B)* for indicated comparisons between groups.
(TIF)
