# Correction: TGFβ-activation by dendritic cells drives Th17 induction and intestinal contractility and augments the expulsion of the parasite *Trichinella spiralis* in mice

**DOI:** 10.1371/journal.ppat.1010063

**Published:** 2021-11-10

**Authors:** Nicola Steel, Aduragbemi A. Faniyi, Sayema Rahman, Stefanie Swietlik, Beata I. Czajkowska, Bethany T. Chan, Alexander Hardgrave, Anthony Steel, Tim D. Sparwasser, Mushref B. Assas, Richard K. Grencis, Mark A. Travis, John J. Worthington

The affiliation for the tenth author is incorrect. Mushref B. Assas is not affiliated with #3 but just with #5: Faculty of Applied Medical Sciences, King AbdulAziz University, Jeddah, Saudi Arabia.
