# Supplementary figures and images for: TGFβ-activation by dendritic cells drives Th17 induction and intestinal contractility and augments the expulsion of the parasite Trichinella spiralis in mice
Source: PLoS Pathog. 2019 Apr 18;15(4):e1007657. doi: 10.1371/journal.ppat.1007657 (PMC6472816; doi:10.1371/journal.ppat.1007657)

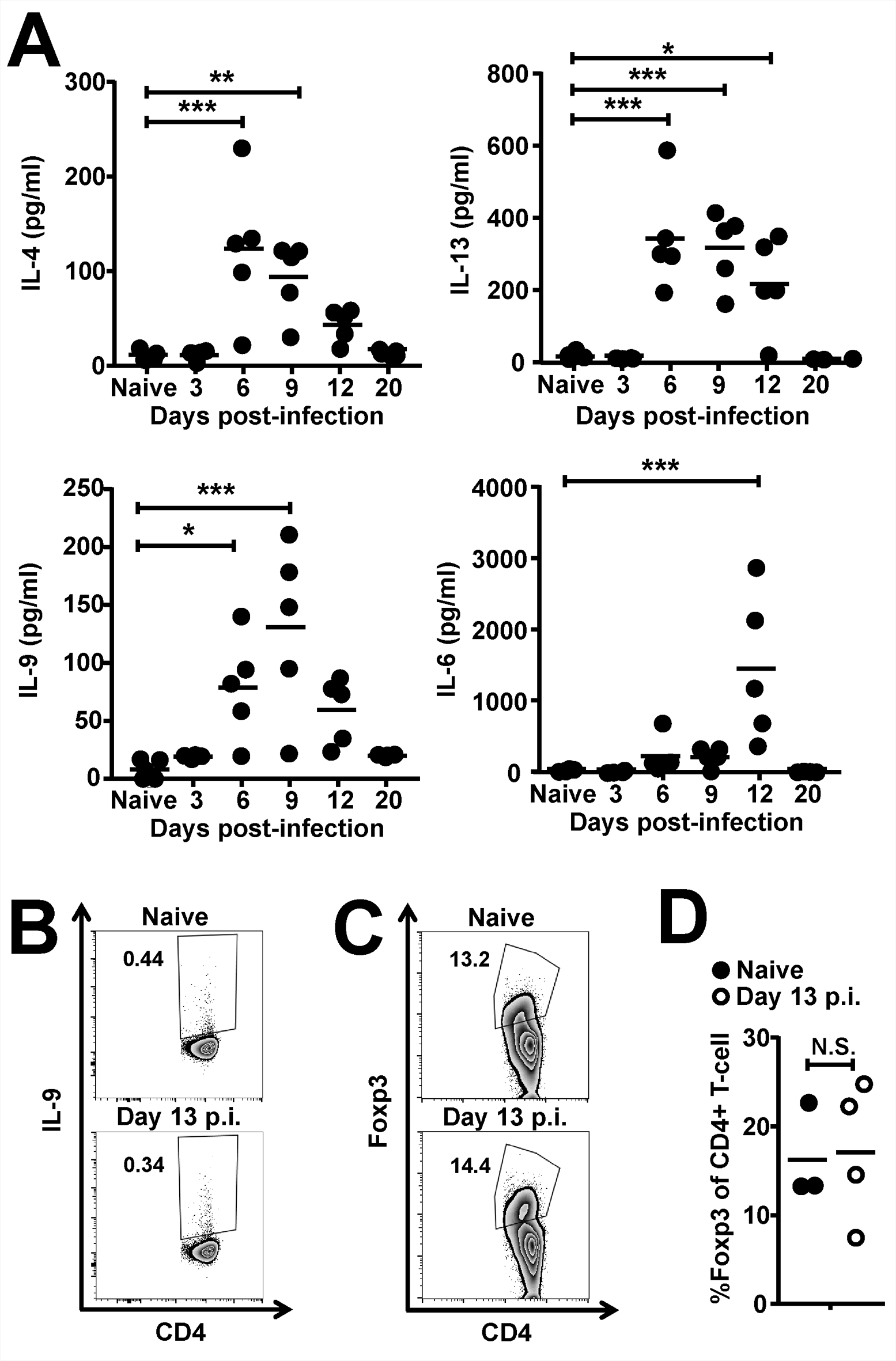

Supplement: S1 Fig — Wild-type C57BL/6 mice were infected with 300 T. spiralis larvae and examined at the indicated time points. (A) IL-4, 13, 6 and 9 cytokine levels from T. spiralis antigen-stimulated mLN cells across the time-course of intestinal infection, determined via cytometric bead array. (B) Representative flow cytometry plots of percentage IL-9 expression in mLN CD4+ T-cells from uninfected and day 13 post-infected mice. (C) Representative flow cytometry plots and (D) Percentage Foxp3 expression in small intestinal lamina propria CD4+ T-cells from uninfected and day 13 post-infected mice. Data (n = 3–5 mice per group) are from two independent experiments performed. *, P<0.05; **, P<0.01; ***, P<0.005; N.S., not significant via Dunnet’s multiple comparison following ANOVA (A) or student’s t-test (D) for the indicated comparisons between groups. (TIF) [file ppat.1007657.s001.tif]

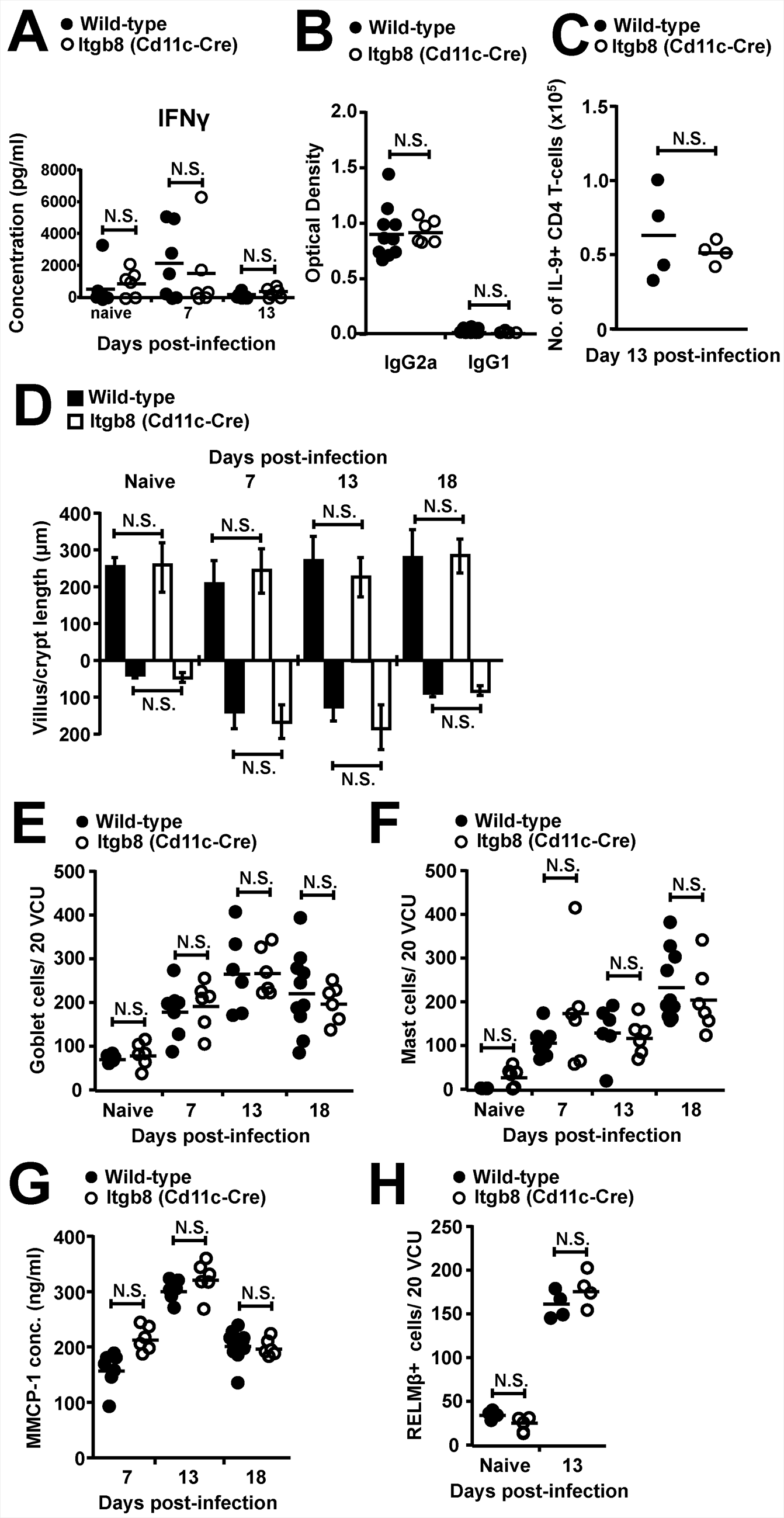

Supplement: S2 Fig — Wild-type and Itgb8 (CD11c-cre) mice were infected with 300 T. spiralis larvae and examined at the indicated time-points post-infection. (A) IFNγ cytokine levels from T. spiralis antigen-stimulated mLN cells from wild-type and Itgb8 (CD11c-cre) mice, determined via ELISA. (B) Parasite-specific serum IgG1 and IgG2a levels in wild-type and Itgb8 (CD11c-cre) mice at day 18 post-infection. (C) Number of IL-9+ CD4 T-cells in the mLN of wild-type and Itgb8 (CD11c-cre) mice at day 13 p.i., assessed via flow cytometry. (D) Villus/crypt lengths assessed via examination of 20 randomly selected VCU in wild-type and Itgb8 (CD11c-cre) mice following infection, quantified via ImageJ software. Number of (E) goblet and (F) mast cells/20 VCU accessed via periodic acid-Schiff’s and toluidine blue histology staining respectively from wild-type and Itgb8 (CD11c-cre) mice. (G) Serum MMCP-1 levels from wild-type and Itgb8 (CD11c-cre) mice following infection, obtained via ELISA. (H) RELMβ+ cells/20VCU from wild-type and Itgb8 (CD11c-cre) mice assessed via immunohistochemistry. All data (n = 4–10 mice per group) are from two independent experiments performed.*, P<0.05; **, P<0.01; ***, P<0.005; N.S., not significant via Bonferonni’s multiple comparison following ANOVA (A), (D), (E-H) or student’s t-test (B),and (C) for the indicated comparisons between groups. (TIF) [file ppat.1007657.s002.tif]

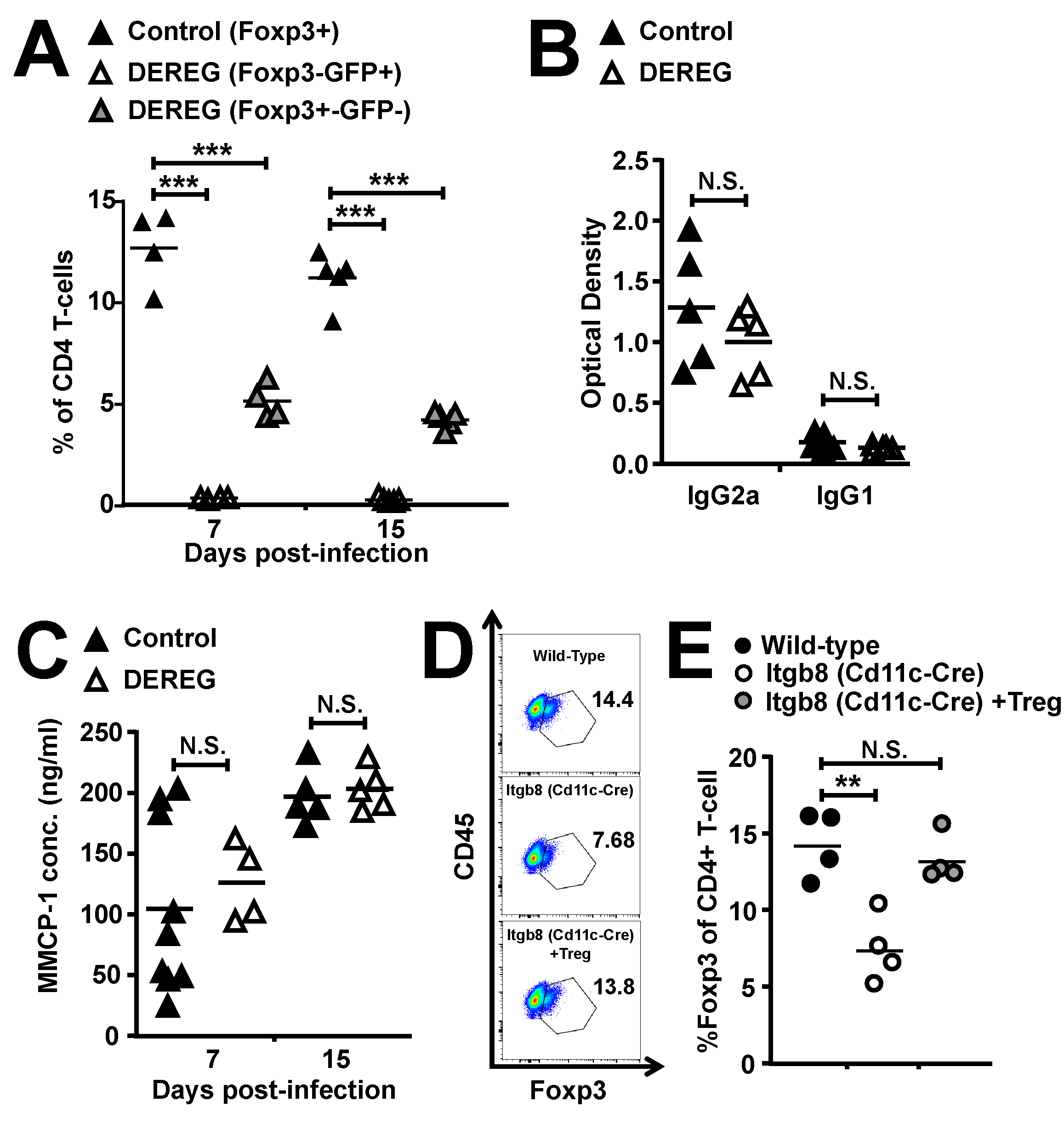

Supplement: S3 Fig — DEREG mice were treated every 2 days with 200 ng diphtheria toxin or PBS (Control) 2 days prior to infection with 300 T. spiralis larvae and examined at the indicated time-points post-infection. (A) The percentage of Foxp3+ CD4 T-cells in the mLN, as assessed via flow cytometry antibody staining and/or Foxp3-GFP reporter. (B) Parasite-specific serum IgG1 and IgG2a levels in Control and DEREG mice at day 15 post-infection, obtained via ELISA. (C) Serum MMCP-1 levels from Control and DEREG mice following infection, obtained via ELISA. Data (n = 4–9 mice per group) are from two independent experiments performed. Wild-type, Itgb8 (CD11c-cre) and Itgb8 (CD11c-cre) mice were adoptively transferred with 1x10^6^ Tregs were infected with 300 T. spiralis larvae 2 days following cell transfer. Representative flow cytometry plots (D) and (E) percentage Foxp3 expression in small intestinal lamina propria CD4+ T-cells from day 13 post-infection. Data (n = 4 mice per group) are from two independent experiments performed. **, P<0.01; ***, P<0.005; N.S., not significant via Dunnet’s multiple comparison following ANOVA (A) and (E), Bonferonni’s multiple comparison following ANOVA (C) and student’s t-test (B) for indicated comparisons between groups. (TIF) [file ppat.1007657.s003.tif]

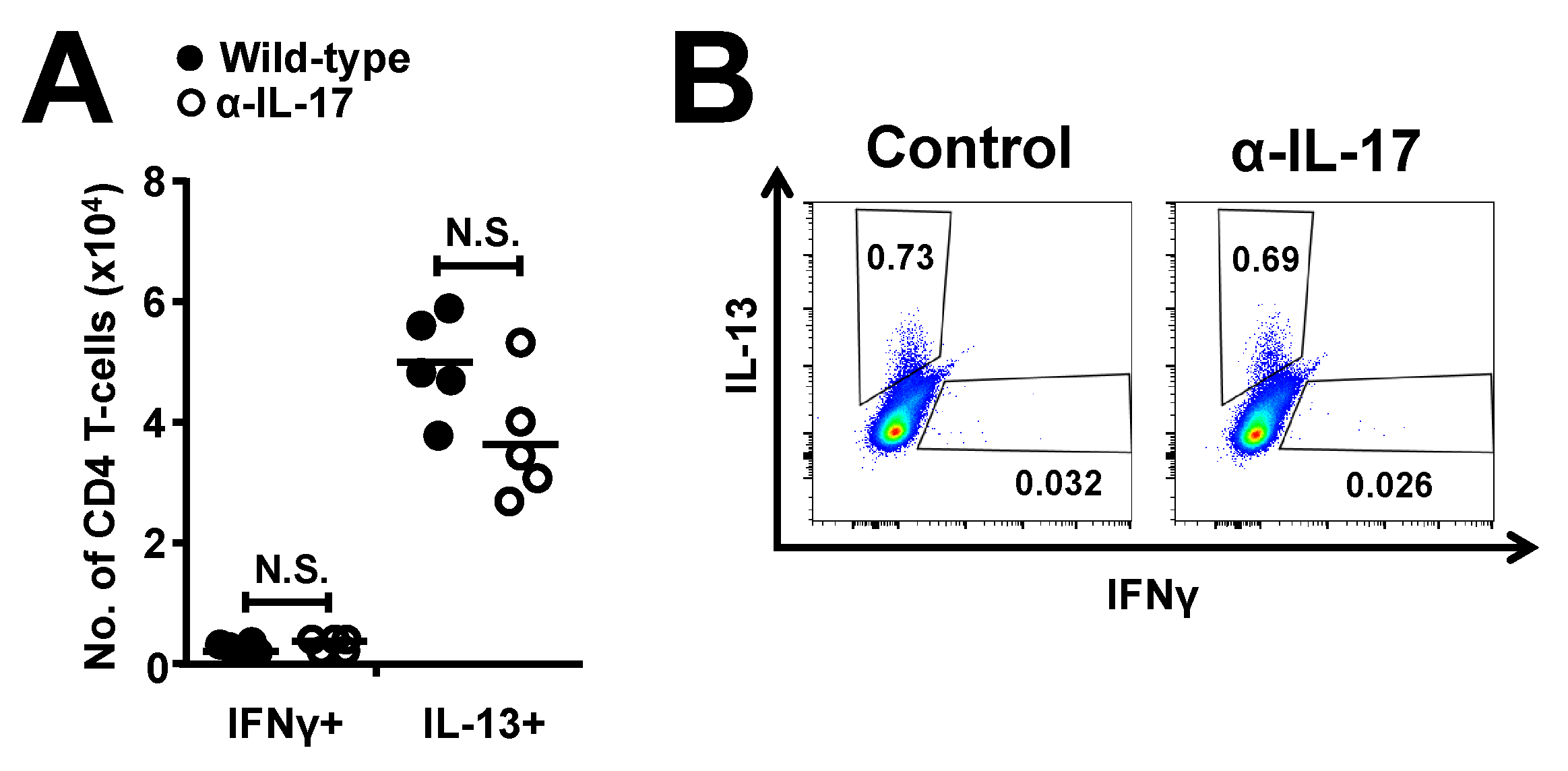

Supplement: S4 Fig — C57BL/6 mice were infected with 300 T. spiralis larvae and treated with 100μg of anti-IL-17 or control antibody (Bio-X-Cell) every 3 days from day 7 post-infection. (A) Number of mLN IFNγ and IL-13 positive CD4+ T-cells and (B) representative flow cytometry plots. Data (n = 5 mice per group) are from two independent experiments performed. N.S., not significant via student’s t-test for indicated comparisons between groups. (TIF) [file ppat.1007657.s004.tif]

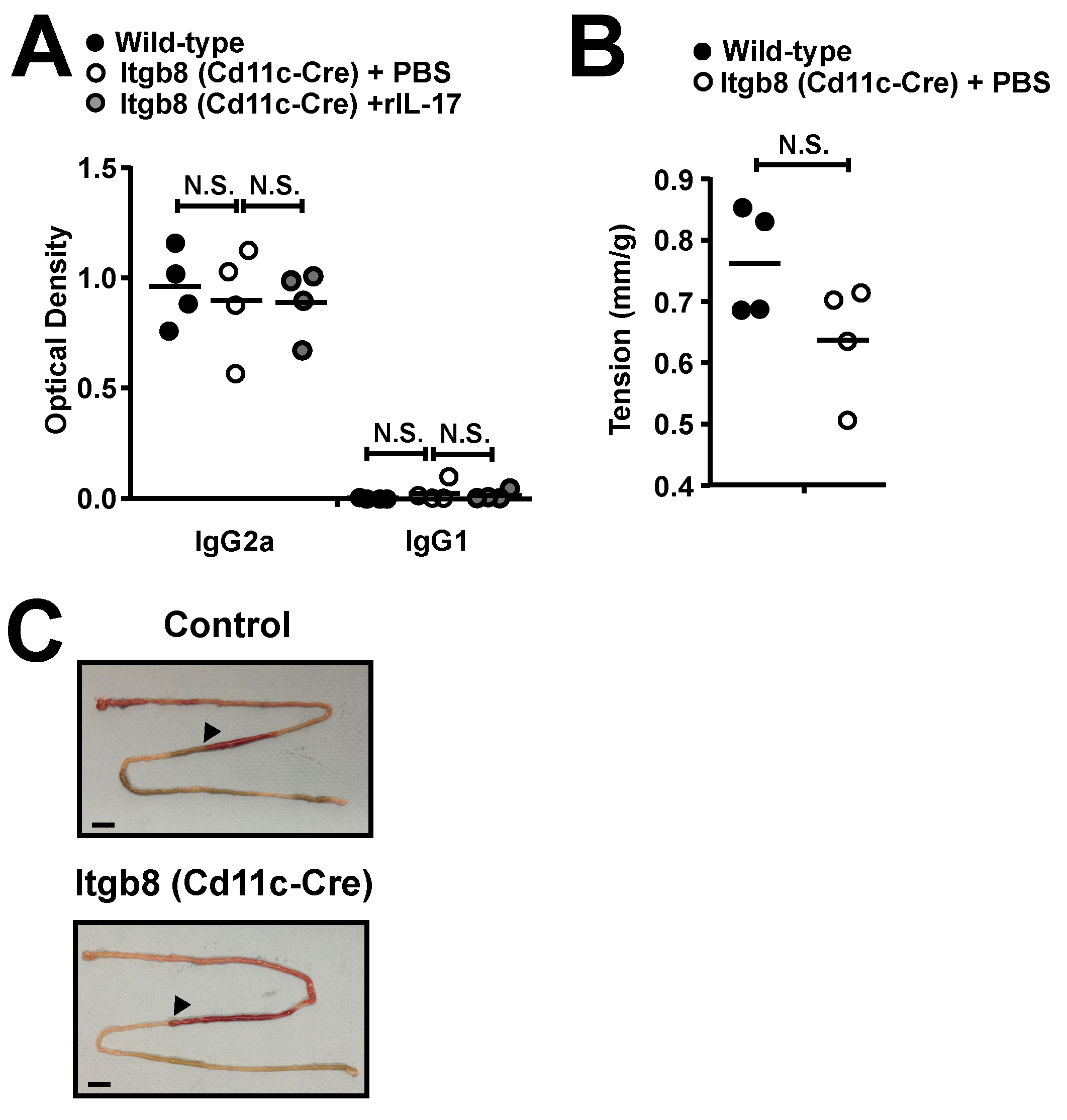

Supplement: S5 Fig — Wild-type and Itgb8 (CD11c-cre) mice were infected with 300 T. spiralis larvae and treated with PBS or 2ug of recombinant IL-17 every 3 days from day 9 post-infection and examined at the indicated time-points post-infection. (A) Parasite-specific serum IgG1 and IgG2a levels in wild-type and Itgb8 (CD11c-cre) PBS or rIL-17 treated mice at day 18 following infection, obtained via ELISA. (B) Base line jejunal longitudinal muscle tension in naïve wild-type and Itgb8 (CD11c-cre) mice in an isolated tissue bath. Chow was removed 12 hrs prior to sacrifice at day 13 and mice received 200μls carmine red in methylcellulose 20 minutes before sacrifice. (C) Representative macroscopic images of wild-type and Itgb8 (CD11c-cre) naïve mice, arrow indicates front of dye and scale bar = 1 cm. Data (n = 4 mice per group) are from two independent experiments performed. N.S., not significant via Bonferonni’s (A) multiple comparison following ANOVA and student’s t-test (B) for indicated comparisons between groups. (TIF) [file ppat.1007657.s005.tif]
